# A Shared, Practical, Interactive Curriculum for Extracorporeal Membrane Oxygenation (SPICE): Time to SPICE It Up!

**DOI:** 10.7759/cureus.114431

**Published:** 2026-08-12

**Authors:** Lauren Alessi, Divya Gupta, Ashley Siems, Peggy Han

**Affiliations:** 1 Department of Pediatric Critical Care Medicine, Corewell Health Helen DeVos Children's Hospital, Michigan State University College of Human Medicine, Grand Rapids, USA; 2 Department of Pediatric Critical Care Medicine, Children's National Hospital, Washington, D.C., USA; 3 Department of Pediatric Critical Care Medicine, Johns Hopkins All Children's Hospital, St. Petersburg, USA; 4 Department of Pediatrics, Division of Pediatric Critical Care Medicine, Stanford University School of Medicine, Lucile Packard Children's Hospital, Palo Alto, USA

**Keywords:** critical care medicine, extracorporeal life support (ecls), extracorporeal membrane oxygenation (ecmo), fellowship, pediatrics, peer mentorship, simulation, veno-arterial extracorporeal membrane oxygenation (va ecmo), veno-venous extracorporeal membrane oxygenation (vv ecmo)

## Abstract

Introduction

Extracorporeal membrane oxygenation (ECMO) is a complex but infrequently used technology that plays a crucial role for patients experiencing cardiopulmonary failure. Formal ECMO education remains inconsistent, with considerable variability in structure and availability. We aimed to implement a shared, practical, and interactive curriculum on ECMO or “SPICE” across multiple institutions.

Methods

Educators collaborated across four different institutions developing an ECMO curriculum that consisted of 15 educational sessions including 10 simulated ECMO emergencies. Three institutions deployed the curriculum over an academic year and a fourth served as a control site. Feasibility was assessed with the percentage of curriculum completed at each site. Effectiveness was assessed by qualitative analysis of fellow response to learning as well as pre- and post- test knowledge assessment and learner confidence.

Results

The curriculum was successfully executed at all three sites, making it a feasible curriculum for any training program that has a simulation center and access to ECMO equipment. Fellows answered an average of 68% (± 10%) correct on medical knowledge assessment pre-curriculum and 64% (± 5%) post-curriculum, which was not statistically different. Average confidence in answers was greater in the SPICE post-curriculum survey versus the control group (2.2±0.2 vs.1.8±0.3, p=0.03). Themes that emerged from the qualitative analysis showed that learners benefited in six domains including self-confidence, knowledge, resources, decision-making, skill practice, and communication.

Conclusions

SPICE is a novel, standardized, and feasible way to teach ECMO skills and increase trainee confidence through hands-on learning.

## Introduction

Extracorporeal membrane oxygenation (ECMO) is a complex technology that plays a crucial role in prolonging life for patients experiencing cardiopulmonary failure. Expertise in ECMO requires a highly specialized skill set, which typically takes years of practice to develop. Thus, teaching ECMO to pediatric trainees presents significant challenges due to its complexity, the low incidence of cases in clinical practice, and the absence of a shared curriculum [1,2]. While some institutions have created didactic sessions or educational boot camps focused on ECMO management, formal ECMO education remains inconsistent across critical care training programs, with considerable variability in structure and availability [1,3,4]. Given that simulation-based ECMO training programs have shown promise in providing effective education [5], there is a pressing need for a shared ECMO curriculum that can be easily implemented across various training programs. Additionally, maintaining frequency and consistency within the curriculum is essential for the retention of skills and knowledge in extracorporeal life support (ECLS) training [6]. In response to these challenges, we aimed to implement a shared, practical, and interactive curriculum on ECMO or “SPICE” across multiple institutions for Pediatric Critical Care Medicine (PCCM) fellows. Our objective was to demonstrate a novel and feasible way to develop a curriculum across institutions using hands-on learning on a high-risk, low-frequency topic. We report the feasibility and effectiveness of implementing SPICE in this multi-institutional descriptive study.

This work was previously presented as a meeting abstract at the 2025 Association of Pediatric Program Directors (APPD) Spring Meeting on March 27, 2025, and at the 36th Annual Extracorporeal Life Support Organization (ELSO) Conference on September 29, 2025.

## Materials and methods

Rooted in Ericsson’s Deliberate Practice Theory, the SPICE curriculum included core topics of ECMO candidacy, oxygenator failure, circuit clot, disseminated intravascular coagulation, cannula dislodgment, air embolus, differential hypoxemia, recirculation, and cardiac tamponade. These cases were taught as high-fidelity mannequin-based simulations using a shared, systematic approach to education, with focus on skills needed to quickly identify the problem, successfully address the emergency, and gather additional resources. Although sharing universal principles of ECMO management, each scenario allowed for practice variability across institutions. All simulation debriefings followed the Promoting Excellence And Reflective Learning in Simulation (PEARLS) framework [7]. Additional teaching sessions included a table-top puzzle activity to arrange pictures of ECMO circuit components as a two-dimensional ECMO circuit, a three-dimensional circuit assembly, an ECMO-specific flashcard exercise, and fill-in-the-blank worksheets as individual or group work.

At each institution, simulation sessions were conducted using existing simulation center personnel, facilities, and equipment, while non-simulation content was delivered during routinely scheduled fellowship educational sessions. Implementation of the curriculum required only existing ECMO program, simulation center, and fellowship educational resources, including ECMO equipment for instruction, and did not generate additional costs beyond those routinely associated with trainee education.

**Figure 1:**
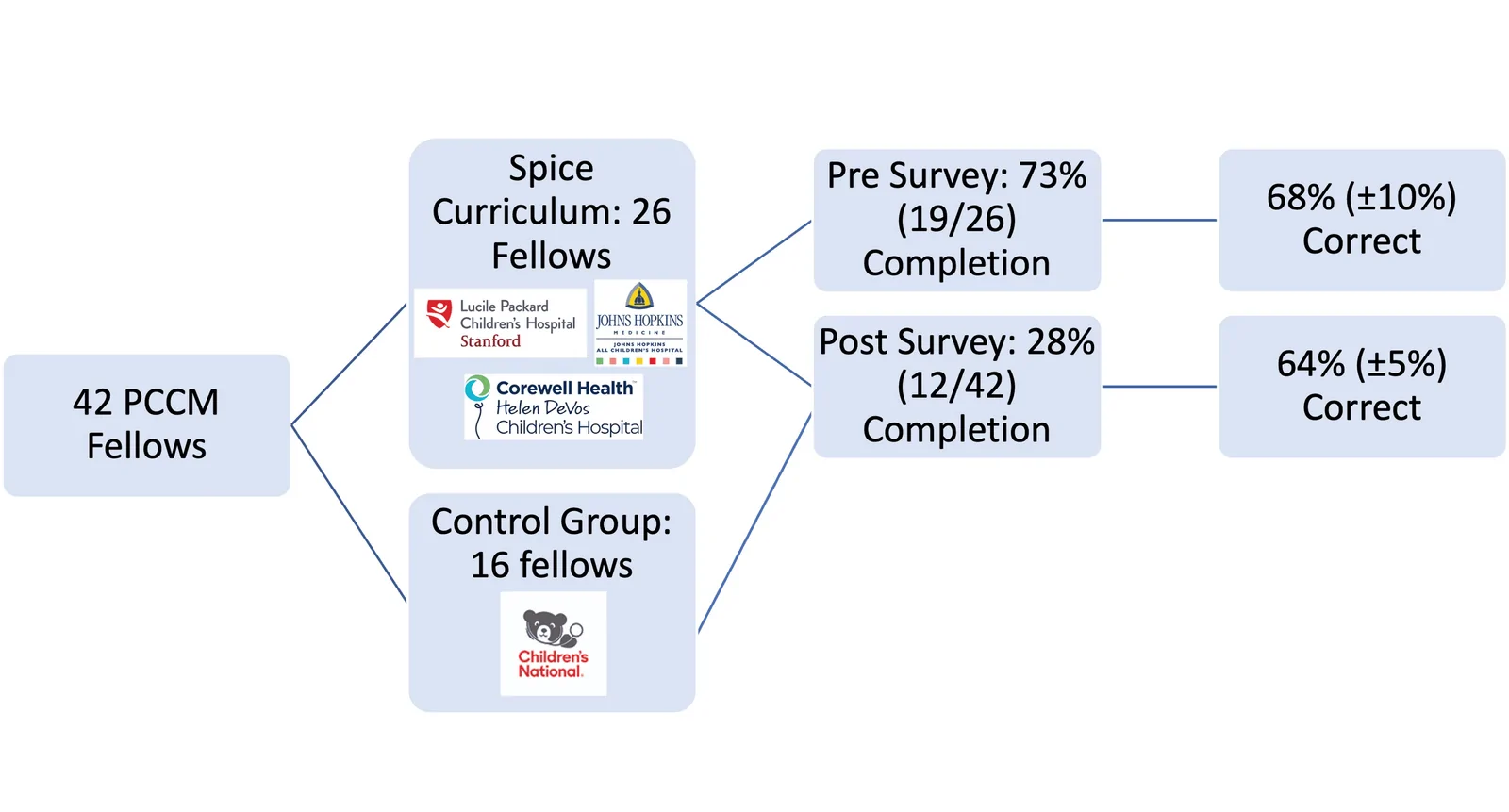
Fellow Participation in SPICE Curriculum SPICE, Shared, Practical, Interactive Curriculum for Extracorporeal Membrane Oxygenation

The curriculum was implemented at three academic pediatric hospitals with fellowship programs of varying size over an academic year (July 2023 to June 2024). A fourth center with a large fellowship program (16 total fellows) served as a control. A total of 42 PCCM fellows enrolled in the study from 2023-2024 (Figure 1). Twenty-six fellows from three institutions participated in a series of five simulation sessions focusing on ECMO initiation and emergencies. Figure 1 shows the division of learners. Table 1 shows the demographic breakdown of each institution. Attendance at each learning session varied across institutions, with a range of 4-8 PCCM fellows, 1-3 faculty members, 1-2 ECMO educators/program coordinators, 1-3 nurses, 0-2 respiratory therapists, 0-3 pharmacists, and occasional hospitalists and advanced practice providers.

**Table 1:** Baseline Characteristics of Participating Institutions *PCCM, Pediatric Critical Care Medicine; ECMO, Extracorporeal Membrane Oxygenation

Domain	Characteristic	Corewell Health Helen DeVos Children's Hospital (HDVCH)	Johns Hopkins-All Children’s Hospital (JHACH)	Lucile Packard Children’s Hospital Stanford (LPCH)	Children’s National Hospital (CNH)
Institutional Profile	Institution type	Academic	Academic	Academic	Academic
	Geographic region	Midwest	Southeast	West	Mid-Atlantic
	ICU model	Separate Cardiac and Pediatric ICU	Separate Cardiac and Pediatric ICU and Thoracic ICU	Separate Cardiac and Pediatric ICU	Separate Cardiac and Pediatric ICU
	Number of ICU Beds	CICU (6 beds) PICU (24 beds)	CICU (22 beds) PICU (28 beds) Thoric Unit (15 beds)	CVICU 36 beds, PICU 36 beds	CICU (26 beds) PICU (48 beds)
ECMO Program	Number of ECMO cases/year	VV 3 VA 9 ECPR 4 Volume 15-20 cases/year	VV 8 VA 33 ECPR 0 Volume 41 cases/year	VV 15 VA 31 ECPR 8 Volume 50-60 cases/year	VV 4 VA 54 ECPR 20 Volume 60-70 cases/year
	Cannulation team	Cardiovascular Surgeons and General Pediatric Surgeons	Cardiovascular and Thoracic Surgeons	Peds Cardiovascular surgeons (CVICU) and General pediatric surgeons (NICU/PICU)	Cardiovascular Surgeons
PCCM* Fellows	Number of PCCM* Fellows	6	6	14	16
	Number of PCCM* Fellows with Cardiology Training	0	0	1	1

Feasibility was assessed by the actual number of educational sessions that were executed at all sites. There were 15 planned didactics that included one interactive lecture, four interactive exercises, and 10 simulations. Across the three institutions, all sessions were completed with the exception of one simulation at site A and one interactive exercise at site B for a completion rate of 96%. The simulation at site A was changed to a discussion case instead of a simulation due to unavailability of a teaching ECMO circuit. The exercise at Site B was transitioned from building an ECMO circuit from paper components and was replaced with a demonstration and assembling an actual circuit. Site A had a total of 15 fellows and an average of six fellows participated in each activity. Site B had a total of five fellows, and an average of four fellows participated in each activity, and site C had a total of six fellows, and an average of five fellows participated in each activity. An average of 60% of all eligible fellows participated in each individual session.

Pre- and post-assessments of knowledge and attitudes were obtained anonymously from SPICE curriculum participants across three institutions. Knowledge questions were originally developed from expert opinions of three pediatric intensivists and assessment of attitudes was determined using a 4-point Likert scale. Cognitive interviews were conducted among five attending physicians (one surgical critical care, four pediatric critical care) for clarity, readability and accuracy of questions prior to use in the study. To avoid recall bias, two separate sets of 25 question-knowledge assessments focusing on ECLS management were created. One set was used for the pre-assessment, and the other set was used for the post-assessment. The post-curriculum questionnaire was administered to the PCCM fellows at a fourth institution to assess their knowledge and confidence regarding ECMO without having participated in SPICE. The knowledge assessments and attitudes were evaluated using descriptive statistics and analyzed differences in confidence levels utilizing the Mann-Whitney U test. Qualitative data in the form of written prose and feedback were obtained from each fellow on the post-curriculum questionnaire. We asked open-ended questions, developed a code system, and collected opinions until we reached set themes. Feedback was organized according to themes that were verified by all authors. 

Free-response qualitative written feedback was also collected from fellows who participated in SPICE. A thematic analysis of the written feedback was performed using previously described qualitative research methods [8,9]. Investigator triangulation was performed by three investigators (D.G., L.A., A.S.) to improve interrater reliability [10]. All discrepancies in codes were resolved after reaching consensus through discussion. After condensing and categorizing codes, emerging themes were identified.

## Results

Pre- and post-SPICE surveys were completed by 73% (19/26) and 28% (12/42) of eligible fellows, respectively. Fellows answered an average of 68% (± 10%) correct on medical knowledge assessment pre-curriculum and 64% (± 5%) post-curriculum, which was not statistically different. Average confidence in answers was greater in the SPICE post-curriculum survey versus control group (2.2±0.2 vs.1.8±0.3, p=0.03) (Table 2). 

**Table 2:** Comparison of Medical Knowledge and Confidence Pre- and Post-SPICE SPICE, Shared, Practical, Interactive Curriculum for Extracorporeal Membrane Oxygenation

	Survey completion	Knowledge (% correct, IQR)	Confidence (Likert scale*)
SPICE Pre-curriculum (26)	(19/26) 73%	68 (58-78)	
SPICE Post-curriculum (26)	(12/42) 28%	64 (59-69)	2.2 ± 0.2
Control Post-curriculum (16)		64 (60-65)	1.8 ± 0.3
Mann-Whitney U test (z score, p value)		225.6 (z = 1.26, p = 0.1)	192 (z = -0.041, p = 0.03)

**Figure 2:**
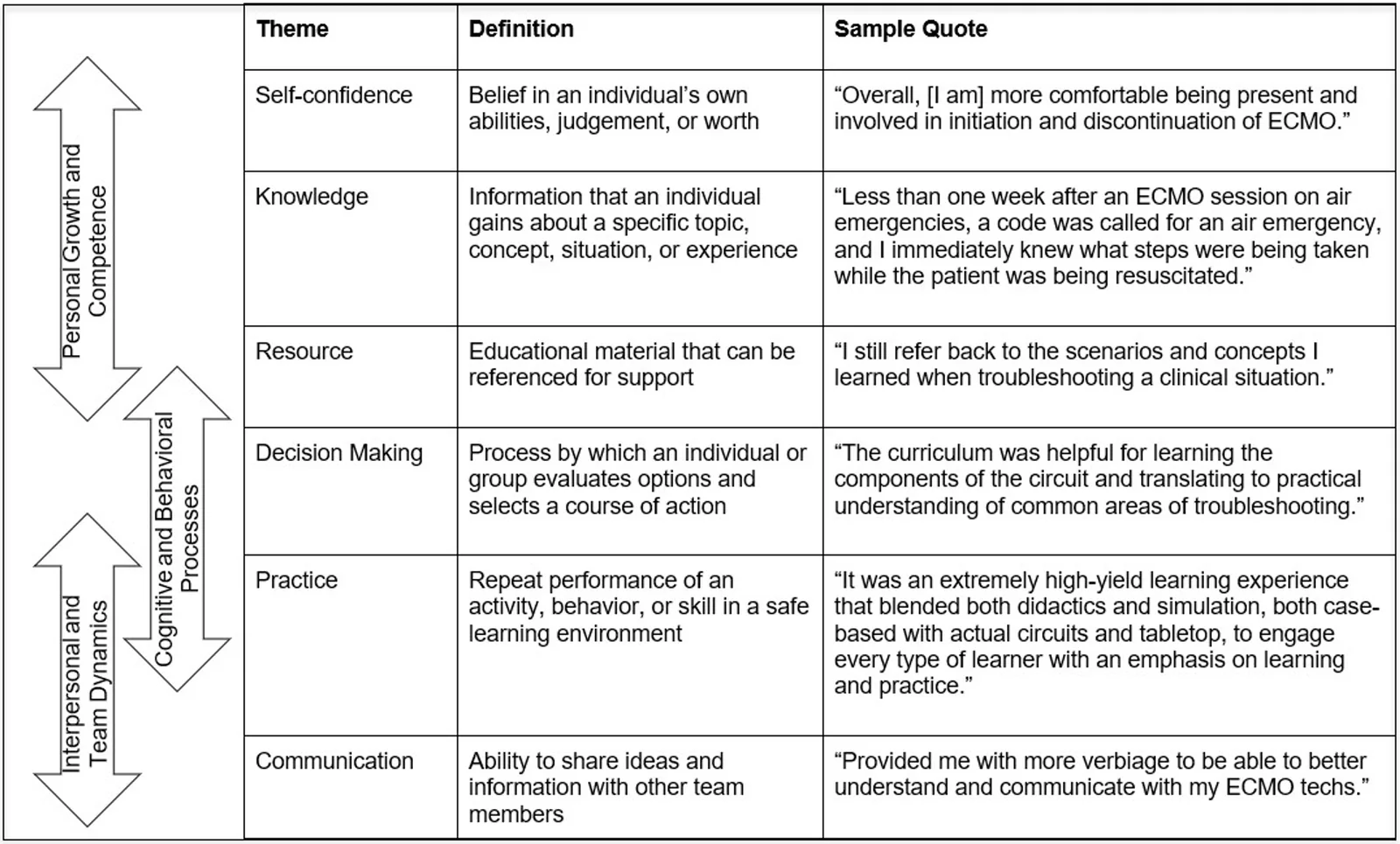
Thematic Categories of Reflective Quotes by Learners ECMO, Extracorporeal Membrane Oxygenation

Qualitative feedback was provided by 12 of 26 fellows via written feedback regarding the curriculum. Six themes emerged (self-confidence, knowledge, decision making, communication, practice, and resource). These themes described fellows’ perception and reflection of the curriculum in three main categories: the curriculum’s contribution to their personal growth and competence (self-confidence, knowledge, resource), improvement in their cognitive and behavioral processes (decision making, practice), and improvement in interpersonal and team dynamics (communication, practice). Figure 2 lists the themes and representative quotes that emerged.

Qualitative feedback was overwhelmingly positive from the PCCM fellows, noting significantly enhanced learning and application of knowledge regarding ECMO at bedside with real patients. Written reflections of the impact of SPICE revealed that all fellows felt more confident in treating patients on ECMO after participation in SPICE. As written by a participant: “SPICE sims have specifically made me feel more agile with troubleshooting issues on a circuit [and] provided me with more verbiage to be better able to understand and communicate with the ECMO perfusionist.” One trainee added, “the ECMO sims will help me to save a life one day as an attending and I highly recommend them for all trainee programs.” The confidence gained through SPICE was not seen in the control group.

## Discussion

SPICE is a novel interactive simulation curriculum that promotes curricular consistency and supports a consistent approach around a low-volume, high-impact clinical modality across institutions. It was developed with input from three different academic institutions and then reviewed by a fourth academic institution. We showed that the curriculum could be executed at any academic hospital with adaptation to each individual hospital system. 

Recently, guidelines for ECMO continuing education and training focus on minimum standards for knowledge, psychomotor, and behavioral competencies, as well as for ECMO simulation, instructor preparation, and standardized certification [5]. While comprehensive for professional hospital teams and systems, the guidelines must be adapted for trainee education. Trainees require structured curricula that teach key concepts, with repetition in both classroom and simulation environments and focused assessments of knowledge and confidence. SPICE allowed for basic concepts to be relayed to PCCM fellows, both in didactic and immersive environments, provided a standard approach to management of ECMO while allowing room for practice discrepancy, and enabled conversation around institutional variables. 

The need for ECMO education for adult critical care medicine trainees has already been established and supports the use of a mixed-methods (simulation and didactic) approach [1]. The incidence of ECMO use in pediatrics is lower than in adult medicine, necessitating an even higher need for curriculum development [11]. Our curriculum helps advance this endeavor by aligning the curriculum across institutions and providing an implementation framework that is easily adaptable to the individual program despite differences in each program (e.g., size, resources, variations in ECMO programs). Establishing minimum standards for training is important considering credentialing in ELS remains inconsistent in the field [12].

The lack of observed improvement in pre- and post-curriculum knowledge assessment scores represents an important limitation of this study. Potential explanations include insufficient validity evidence for the knowledge assessment tool and a sample size that may have been underpowered to detect changes in learner knowledge following curricular implementation. On retrospective review by the authors, there was concern that the post-curriculum questions were more difficult compared to pre-curriculum questions, despite being randomly divided. This difference in questions could explain the lack of statistically significant increase in knowledge when comparing pre- and post-curricular data. Additionally, our study was limited by a low response rate, which may reflect a non-response bias. The questions were also developed to assess general ECMO knowledge and not specifically created to address the goals and objectives of the curriculum. Previous studies have shown that simulation-based ECMO education can improve time to critical task performance [13], and future directions could include measuring performance in a similar way with this curriculum.

The qualitative analysis shows a perceived benefit in skills, comfort, and knowledge gained regardless of the lack of improvement on the knowledge assessment. In recent years, new ECMO fellowship programs have been added to the academic landscape to promote competency in this topic. We postulate that this immersive ECMO curriculum may provide sufficient ECMO experience to offset the extra year of training, potentially reducing costs for trainees and maintaining a competent workforce. Whether augmenting ECMO education during routine training is equivocal to adding additional training years for trainees is an important area of future investigation.

An additional benefit of this curriculum is that it is multi-modal. Previous studies show the benefit of simulation in ECMO training programs [13-15], and this program incorporates not just simulation curricula but also hands-on learning with ECMO equipment and interactive activities. The curriculum is also spread throughout the course of an academic year, which has shown better retention of skills than annual training [15,16].

SPICE importantly created a network of peer mentorship among each institution’s faculty leaders. In the context of a shifting political and academic landscape, particularly amid budget constraints, it is essential to explore innovative collaborative approaches to enhance simulation-based curricular interventions. Although ECMO is regarded as a rare occurrence in pediatrics, offering frequent, high-quality training provides a viable alternative for fostering connections, promoting learner development, and ensuring safe patient care. By sharing a freely accessible curriculum, we may establish a common educational framework and harmonize ECMO training across training programs. This collaboration not only strengthens the educational experience but also builds networks among institutions that might otherwise remain disconnected.

## Conclusions

In conclusion, SPICE is a novel and feasible way to teach PCCM fellows core ECMO skills and increase trainee confidence through hands-on learning. Additionally, dissemination of an ECMO simulation curriculum is a way to foster collaboration for educational endeavors and promote peer mentorship.
